# Interneurons of fan-shaped body promote arousal in *Drosophila*

**DOI:** 10.1371/journal.pone.0277918

**Published:** 2022-11-21

**Authors:** Yoshiaki S. Kato, Jun Tomita, Kazuhiko Kume

**Affiliations:** Department of Neuropharmacology, Graduate School of Pharmaceutical Sciences, Nagoya City University, Nagoya, Japan; Biomedical Sciences Research Center Alexander Fleming, GREECE

## Abstract

Sleep is required to maintain physiological functions and is widely conserved across species. To understand the sleep-regulatory mechanisms, sleep-regulating genes and neuronal circuits are studied in various animal species. In the sleep-regulatory neuronal circuits in *Drosophila melanogaster*, the dorsal fan-shaped body (dFB) is a major sleep-promoting region. However, other sleep-regulating neuronal circuits were not well identified. We recently found that arousal-promoting T1 dopamine neurons, interneurons of protocerebral bridge (PB) neurons, and PB neurons innervating the ventral part of the FB form a sleep-regulatory circuit, which we named “the PB-FB pathway”. In the exploration of other sleep-regulatory circuits, we found that activation of FB interneurons, also known as pontine neurons, promoted arousal. We then found that FB interneurons had possible connections with the PB-FB pathway and dFB neurons. Ca^2+^ imaging revealed that FB interneurons received excitatory signals from the PB-FB pathway. We also demonstrated the possible role of FB interneurons to regulate dFB neurons. These results suggested the role of FB interneurons in sleep regulation.

## Introduction

Sleep is essential for many physiological functions and is conserved across mammals and invertebrates. Although sleep plays an important role in our lives, sleep regulation mechanisms have not been completely elucidated. To understand the mechanisms of sleep regulation, it is critical to unravel sleep-regulatory genes and neuronal circuits. *Drosophila melanogaster* has been widely used to study the mechanisms of sleep regulation [1, 2]. A large number of sleep-regulatory genes have been reported in *Drosophila* [3–5]. However, the specific brain regions or neural circuit where those genes function to regulate sleep are not well identified. This makes the elucidation of molecular mechanisms of sleep regulation difficult.

Regarding sleep-regulatory circuits, the central complex is of particular interest. The central complex is divided into four regions: the protocerebral bridge (PB), the fan-shaped body (FB), the ellipsoid body (EB), and the noduli (NO). In particular, the dorsal FB (dFB) neurons promote sleep [6], the EB R5 neurons regulate sleep homeostasis [7], and these neurons interact with each other [8]. In previous studies, we found that dopamine is a major regulator of arousal and identified a dopamine pathway from PPM3 to FB [9, 10]. Recently, we found that T1 dopaminergic neurons (arousal-promoting), R59E08 neurons (neurons labeled by R59E08-Gal4 driver, which label sleep-promoting PB interneurons), and R52B10 neurons (neurons labeled by R52B10-Gal4 driver, which label arousal-promoting P-FN neurons) that project from the PB to the ventral FB and the NO form a sleep-regulatory circuit, hereafter referred to as “the PB-FB pathway” [11].

In this manuscript, we explored the sleep-regulatory circuits in the central complex and found that FB interneurons, also known as pontine neurons, promoted arousal. We also found that R52B10 neurons formed an excitatory connection with FB interneurons. In addition, we found that FB interneurons likely formed close associations with dFB neurons which regulate sleep.

## Materials and methods

### Fly strains and rearing conditions

Fruit flies (*Drosophila melanogaster*) were raised at 25°C in 50–60% relative humidity on standard medium containing cornmeal, yeast, glucose, wheat germ, and agar, as described before [9]. They were maintained under a 12-h light: dark (LD) cycle. In this study, we used *R52B10-Gal4* (BDSC stock number 38820), *R23E10-Gal4* (49032), *R23E10-LexA* (52693), *R52B10-LexA* (52826), *UAS-mCD8*::*GFP* (5130), *tub-Gal80*^*ts*^ (7019), *UAS-GCaMP6s* (42746), *UAS-DenMark*, *syt*.*eGFP* (33064), *LexAop-P2X2* (76030), *hDeltaC-Gal4* (75925), and *vDeltaB*, *C*, *D-Gal4* (93172) from the Bloomington *Drosophila* Stock Center, and *NP2320-Gal4* (DGRC stock number 104157) from the *Drosophila* Genetics Resource Center. *UAS-dTrpA1* [12] was a gift from Dr. Julie H. Simpson. *Cha*-*Gal80* [13] was from Dr. Takaomi Sakai. *UAS-CD4*::*spGFP1-10*, *LexAop- CD4*::*spGFP11* were from Dr. Kristin Scott. *UAS-Kir2*.*1* [14] was from Dr. Richard A. Baines. *R52B10-Gal4*, *R23E10-Gal4*, *NP2320-Gal4*, and *UAS-dTrpA1* are backcrossed at least 5 times to the control strain (*w*^*1118*^). Male flies were used in all experiments.

### Locomotor activity and sleep analysis

The locomotor activity of individual flies was measured for 1-min intervals using the *Drosophila* activity monitoring system (TriKinetics, Waltham, MA, USA) as described previously [9]. The flies were placed individually in glass tubes (length, 65 mm; interior diameter, 3 mm) containing 1% agar and 5% sucrose food at one end and were entrained for at least 3 days to LD conditions before changing to constant dark (DD) conditions. Activity data were collected continuously under LD and DD conditions. Because sleep in the daytime under LD conditions is partly regulated by light-induced suppression of locomotor activity [9], results from DD conditions (day 2–4 of the DD) are mainly shown. Based on previous reports, sleep in *Drosophila* was defined as continuous immobile periods lasting 5 min or longer. The total activity counts and the total amount of sleep time in DD conditions were analyzed using Microsoft (Redmond, WA, USA) Excel-based software or R (R Core Team, 2020, https://www.r-project.org).

### Immunohistochemistry and image acquisition

Whole-mount immunofluorescence staining of adult *Drosophila* brains (Figs 2B and 3C) was performed as previously described [15]. Other samples were imaged without staining. Adult fly brains were dissected in PBS and fixed in 4% PFA in PBS for 20 min at room temperature. The brains were then washed three times in 0.3% PBS-T for 20 min. After washing, the samples were blocked in 5% normal goat serum (NGS) at 4°C overnight. The next day, the NGS solution was replaced by primary antibody solution in 5% NGS and incubated at 4°C for 1 to 2 days. After washing three times, the samples were incubated in secondary antibody solution in 5% NGS at 4°C for 1 to 2 days. After washing three times, the brains were mounted using PermaFluor (Funakoshi). In the GFP reconstitution across synaptic partners (GRASP) experiment, monoclonal anti-GFP (G10362, ThermoFisher) at 1:100 dilution and anti-nc82 (Developmental Studies Hybridoma Bank, University of Iowa) at 1:100 were used as the primary antibodies. Alexa Fluor 488 goat anti-rabbit IgG (A11034, Invitrogen) and Alexa Fluor 568 goat anti-mouse IgG (A11004, Invitrogen) at 1:1000 were used as secondary antibodies. All brain tissues were imaged using a ZEISS LSM 800 confocal microscope (ZEISS).

### Ca^2+^ imaging

Male flies were dissected in calcium-free adult hemolymph-like saline consisting of 108 mM NaCl, 5 mM KCl, 8.2 mM MgCl_2_, 4 mM NaHCO_3_, 1 mM NaH_2_PO_4_, 5 mM trehalose, 10 mM sucrose, and 5 mM HEPES (pH 7.5). The isolated brains were placed at the bottom of a well of an 8-well plate (ibidi, Germany) beneath the adult hemolymph-like saline. All imaging was performed using a ZEISS LSM 800. To reduce the effect of the z-plane drift, the pinhole was adjusted to 105 μm. All images were taken using a 10x objective lens. Time-series images were collected for 180 s at 1 Hz. After taking baseline images for 60 s, 25 mM ATP was applied by bath application using a pipette. A region of interest (ROI) was determined based on the GCaMP baseline signal on the FB neuropile and drawn around the target structure using Fiji software (https://fiji.sc). The fluorescence signal in each ROI was analyzed using Fiji software. The transition in fluorescence was calculated following this formula: ΔF = Ft-F0/F0 (Ft: fluorescence at time point n; F0: fluorescence at time 0).

### Experimental design and statistical analysis

Data were analyzed as described in each Figure Legend using Microsoft Excel and R. The number of flies used in the experiments is also described in Figure Legends.

## Results

### Activation of FB interneurons promotes arousal

**Fig 1:**
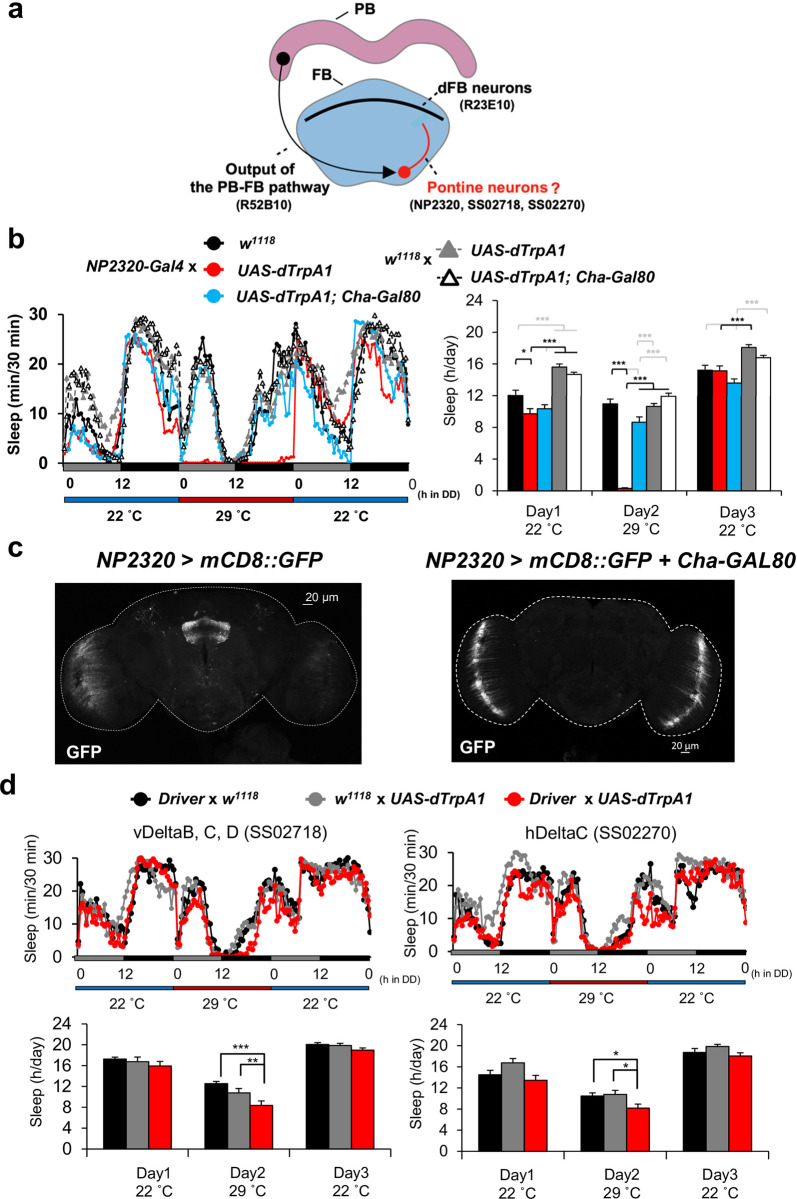
Cholinergic FB interneurons promote arousal. (a) Schematic diagram of the hypothesis of the sleep-regulatory circuit. The names of driver lines are described in the parentheses. (b) right: Sleep profile of each genotype (n = 20, 32, 21, 32, 32 respectively). Thermo-genetic activation by *dTrpA1* occurs at 29 ˚C but not at 22 ˚C. left: Quantification of the sleep time. Data are presented as mean + SEM. one-way ANOVA with a Tukey-Kramer HSD test was used. * P < 0.05, ** P < 0.01, *** P < 0.001 (c) GFP expression pattern of *NP2320-Gal4* with and without *Cha-Gal80*. (d) top: Sleep profile of each genotype (n = 16, 16, 16, 14, 16, 16 respectively). Thermo-genetic activation by *dTrpA1* occurs at 29 ˚C but not at 22 ˚C. bottom: Quantification of the sleep time. Data are presented as mean + SEM. one-way ANOVA with a Tukey-Kramer HSD test was used. * P < 0.05, ** P < 0.01, *** P < 0.001.

To explore novel sleep-regulatory circuits, we focused on the FB interneurons since the output of the PB-FB pathway project to the ventral FB and sleep-promoting dFB neurons arborize in the dorsal FB (Fig 1A). We used a driver line *NP2320-Gal4* which was reported to label FB interneurons [16–19]. To investigate the role of FB interneurons in sleep regulation, we performed a transient thermo-genetic activation of FB interneurons using the thermo-activatable cation channel dTrpA1, which is more active at 29°C and less active at 22°C [12]. Flies were transferred from 22°C to 29°C to activate the FB interneurons and then returned to 22°C. As a result, a significant decrease in sleep time was found on Day 2 at 29°C (Fig 1B). Moreover, *Cha-Gal80*, which inhibits Gal4 in the cholinergic neurons, almost completely suppressed this phenotype (Fig 1B). These results indicated that cholinergic FB interneurons promote arousal. To confirm that FB interneurons are cholinergic, we investigated whether *Cha-Gal80* suppressed GFP expression in the FB. We found that GFP signals in the FB were decreased compared to the control (Fig 1C). We next tested the arousal-promoting effect of FB interneurons using more specific drivers. We used two split-Gal4 driver lines, namely SS02718 and SS02270, which label specific types of FB interneurons named vDeltaB, C, D, and hDeltaC neurons [20] (S1 Fig). Using these drivers, we performed the same experiment as Fig 1B and confirmed the arousal-promoting phenotype in both drivers (Fig 1D). These results indicated that activation of FB interneurons promotes arousal.

### FB interneurons receive excitatory input from the output neurons of the PB-FB pathway

**Fig 2:**
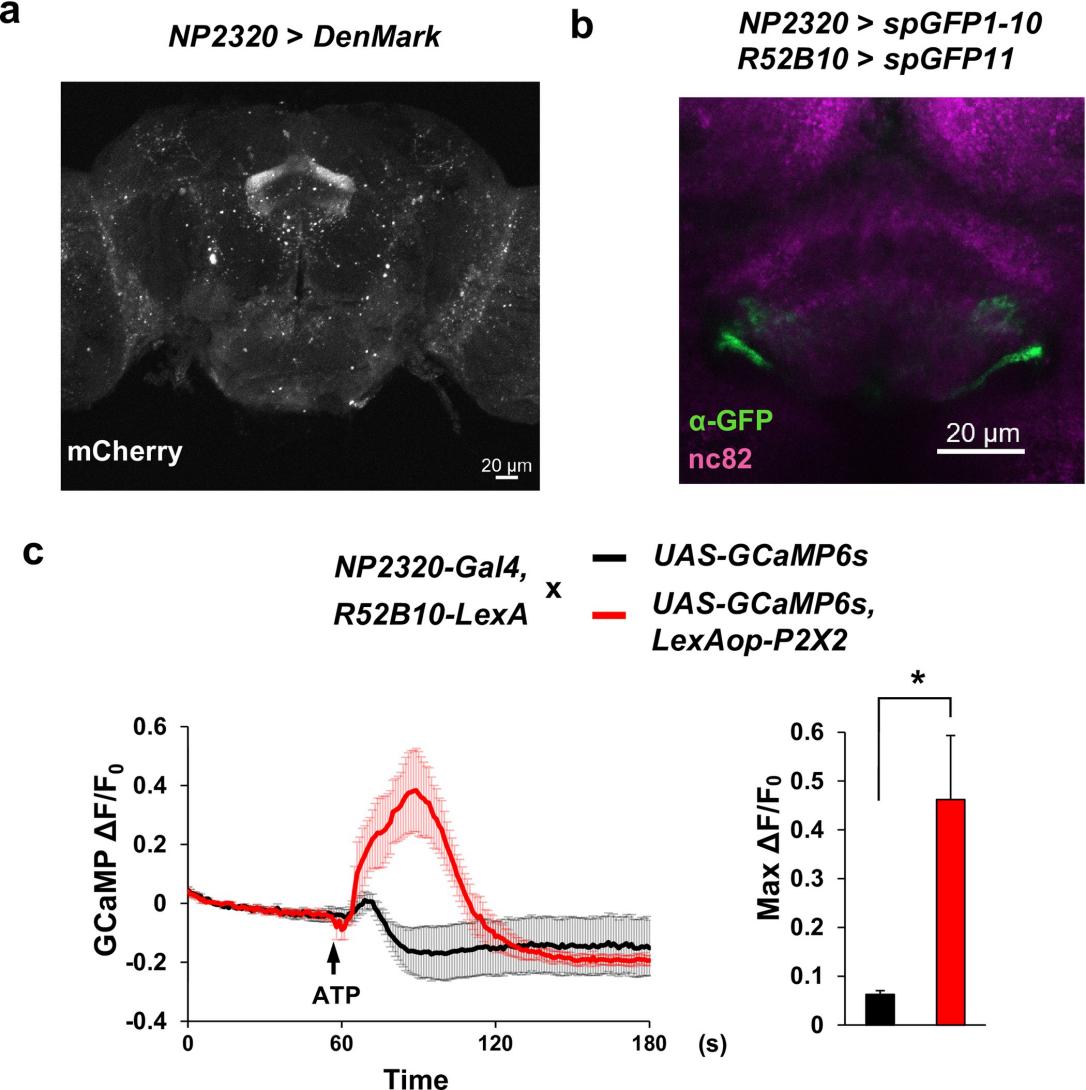
FB interneurons and R52B10 neurons have excitatory connections. (a) Expression pattern of the dendrite marker *DenMark* in FB interneurons. (b) GRASP signals between R52B10 neurons and FB interneurons labeled by NP2320. (c) left: The trace of the GCaMP signals change. R52B10 neurons that express P2X2 were activated by ATP and the GCaMP signals in FB interneurons were measured. The control group (black) and experimental group (red) were both n = 5. right: Quantification of Max ΔF/F_0_. Data are presented as mean + SEM. Two-sided Welch’s *t-*test was used. * P < 0.05.

Next, we asked about the connection between the output neurons of the PB-FB pathway and FB interneurons. We first labeled the dendrites of FB interneurons using the dendrite marker DenMark, which is mCherry-tagged hybrid protein of mammalian ICAM5/Telencephalin [21]. We found that DenMark was expressed in both the dorsal and ventral FB (Fig 2A). We then performed GRASP experiment [22, 23] to confirm the connections between the output neurons of the PB-FB pathway and FB interneurons. We expressed individual split GFP components (GFP1-10 and GFP11) in NP2320 and R52B10 which label the output neurons of the PB-FB pathway and tested whether reconstituted GFP signals were observed. We found that reconstituted GFP signals were in the ventral FB (Fig 2B). This result suggested that FB interneurons are likely to receive signals from R52B10 neurons. To confirm the functional connection, we conducted *ex vivo* Ca^2+^ imaging. We expressed the ATP-gated cation channel P2X2 [24] in R52B10 neurons and the Ca^2+^ indicator GCaMP6s [25] in FB interneurons. By applying ATP to the isolated fly brain in the chamber, R52B10 neurons are activated by P2X2 and if R52B10 neurons and FB interneurons have functional connections, the change of GCaMP signals is observed. As a result, we found a substantial increase in the GCaMP signals in the FB after ATP was applied to the isolated fly brain (Fig 2C and S1 Movie; P = 0.038; two-sided Welch’s *t-test*). Taken together, these results indicated that FB interneurons receive excitatory signals from R52B10 neurons.

### FB interneurons form close associations with dFB neurons

**Fig 3:**
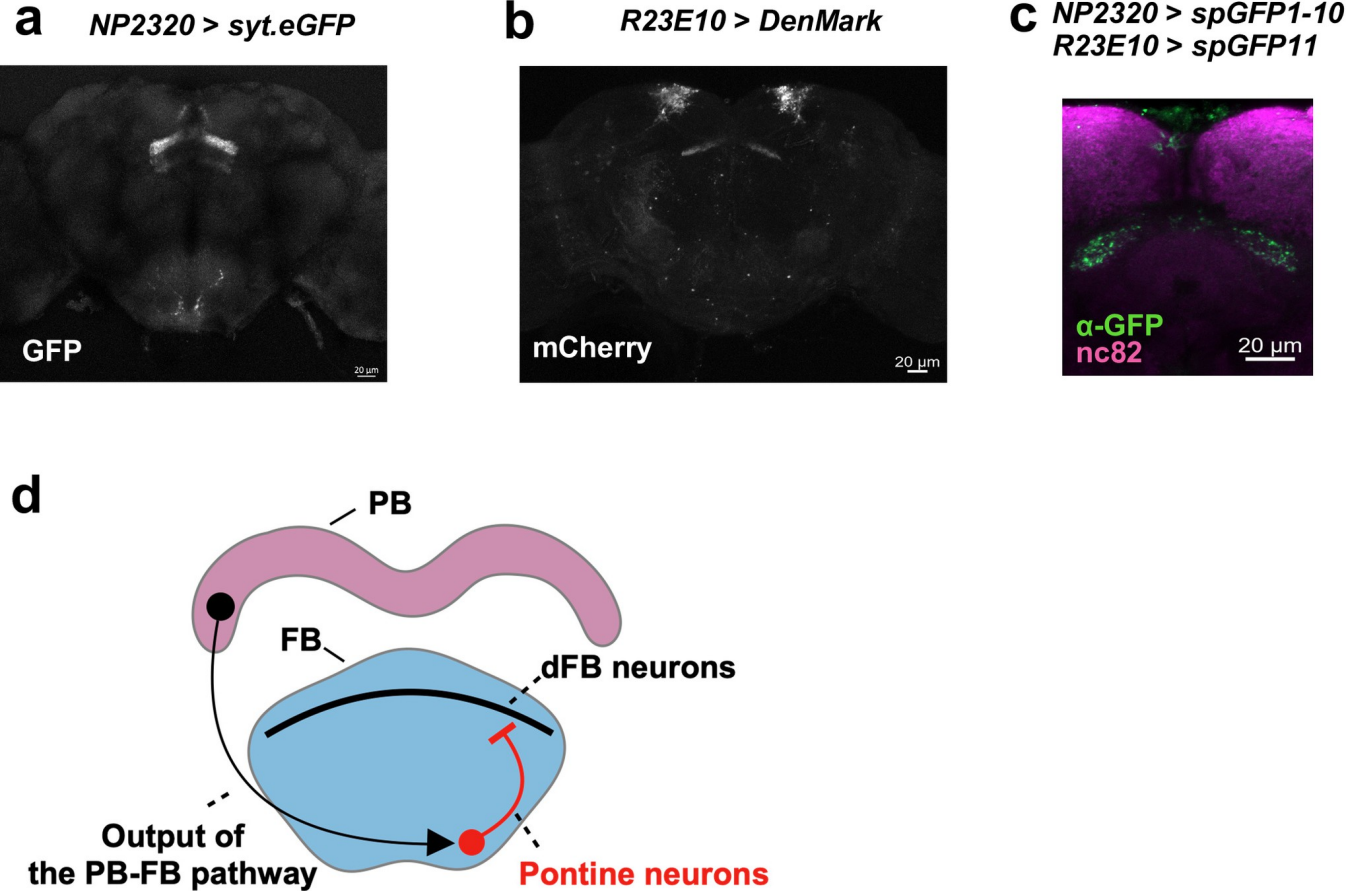
FB interneurons and dFB neurons form close associations. (a) Expression pattern of *syt*.*eGFP* in NP2320-labeled neurons. (b) Expression pattern of *DenMark* in R23E10-labeled neurons. (c) GRASP signals between dFB neurons labeled by R23E10 and FB interneurons labeled by NP2320. (d) Schematic diagram of the possible sleep-regulatory circuit.

To investigate the post-synaptic targets of FB interneurons, we expressed syt.eGFP, an axon terminal marker, in FB interneurons. We found that the axon terminals of FB interneurons were arborized in the dorsal FB (Fig 3A). From this result, we hypothesized that one of the post-synaptic partners of FB interneurons is the sleep-promoting dFB neurons. Then we expressed DenMark in dFB neurons with *R23E10-Gal4*. We found the dendrites of dFB neurons also arborized in the dorsal FB (Fig 3B). We next conducted GRASP experiment to investigate whether FB interneurons form close associations with dFB neurons and observed GRASP positive signals in the dorsal FB (Fig 3C). These results suggested that FB interneurons form close associations with dFB neurons.

## Discussion

This study reports a novel sleep-regulatory pathway that promotes arousal. We first focused on FB interneurons and found that cholinergic FB interneurons promoted arousal (Fig 1B and 1C). We confirmed the arousal-promoting effect of FB interneurons by using more specific drivers (Fig 1D). These drivers label FB interneurons which receive input from P-FN neurons and send output to dFB neurons. There should be other FB interneurons that do not have a connection with P-FN neurons or dFB neurons. It means that FB interneurons labeled by two split drivers are only a part of FB interneurons. Therefore we considered that the weaker effects in Fig 1D compared to Fig 1B were due to the smaller number of neurons labeled by split-Gal4 lines than NP2320, not the effect of neurons other than FB interneurons. In Fig 1B and 1D, we didn’t observe a clear sleep rebound after neuronal activation. In the previous study [11], we used R52B10-Gal4 which is reported to drive sleep rebound in the female fly [7]. We observed a clear sleep rebound in female flies but not in male flies. These results indicated that there is a sex difference in the regulation of sleep rebound, at least, in R52B10 neurons. In this study, we used only male flies and this could be one of the reasons why we didn’t observe a clear sleep rebound. We next asked about the relationship between FB interneurons and known sleep-regulatory circuits. We performed GRASP and Ca^2+^ experiments and showed that FB interneurons and R52B10 neurons which label the output neurons of the PB-FB pathway were anatomically and functionally connected (Fig 2). Although we did not exclude the possibility of other pathways downstream to R52B10, we demonstrated clearly that FB interneurons are one of the downstream to R52B10. Further study will show the impact size of the connection between them in sleep regulation. To investigate the postsynaptic partners of FB interneurons, we also conducted GRASP experiment. We found that FB interneurons and dFB neurons labeled by R23E10 formed close associations (Fig 3A–3C). Besides, according to the connectome paper [26] and connectome dataset [27], vDeltaB, C, D, and hDeltaC receive input from P-FN neurons and send output to FB tangential neurons which arborize in layers 6 and 7. This information also supported the idea that R52B10 neurons including P-FN neurons, FB interneurons like vDeltaB, C, D, and hDeltaC neurons, and dFB neurons that are consisted of FB tangential neurons which arborize in layers 6 and 7 form a neuronal circuit. Future study will clarify their functional connection and the role of this circuit in sleep regulation. Furthermore, a previous study showed that neurons that project to the ventral FB (vFB neurons) promote sleep and mediate consolidation of long-term memory [28]. Since axon terminals and dendrites of FB interneurons arborize in both the dorsal and ventral FB, there would be interactions between dFB and vFB neurons via FB interneurons. Further research will clarify the functional relationship between these neurons.

According to previous reports, FB interneurons regulate optomotor behavior and express tachykinin, a neuropeptide that regulates aggression [17, 19, 29]. Additionally, T1 dopamine neurons, which are upstream of R52B10 neurons [11], regulate aggression as well [30]. Besides, P2 neurons, which include FB interneurons, regulate chronic isolation evoked sleep loss [31]. Moreover, courtship-regulator P1 neurons activate T1 neurons and modulate sleep/courtship balance based on the nutritional status [32]. Taking all the information mentioned above into account, we consider that arousal signals related to aggression, courtship, nutrition, and vision converge into the PB-FB pathway including FB interneurons to regulate arousal. Further studies should clarify the role of these arousal signals on the PB-FB pathway and FB interneurons in sleep regulation.

In conclusion, our results provided possible sleep-regulatory neurons that may connect with the PB-FB pathway and dFB neurons. We hypothesized that arousal signals are sent from the PB-FB pathway to FB interneurons, inhibit dFB neurons via inhibitory signals, and regulate sleep (Fig 3D).

## Supporting information

S1 Fig Expression pattern of two split-GAL4 drivers of FB interneurons. (a) Expression pattern of SS02718 and (b) SS02270 (from Janelia FlyLight Split-GAL4 Driver Collection).
(TIF)

S1 File (ZIP)

S1 Movie (AVI)
