# Supplementary figures and images for: Interneurons of fan-shaped body promote arousal in Drosophila
Source: PLoS One. 2022 Nov 21;17(11):e0277918. doi: 10.1371/journal.pone.0277918 (PMC9678257; doi:10.1371/journal.pone.0277918)

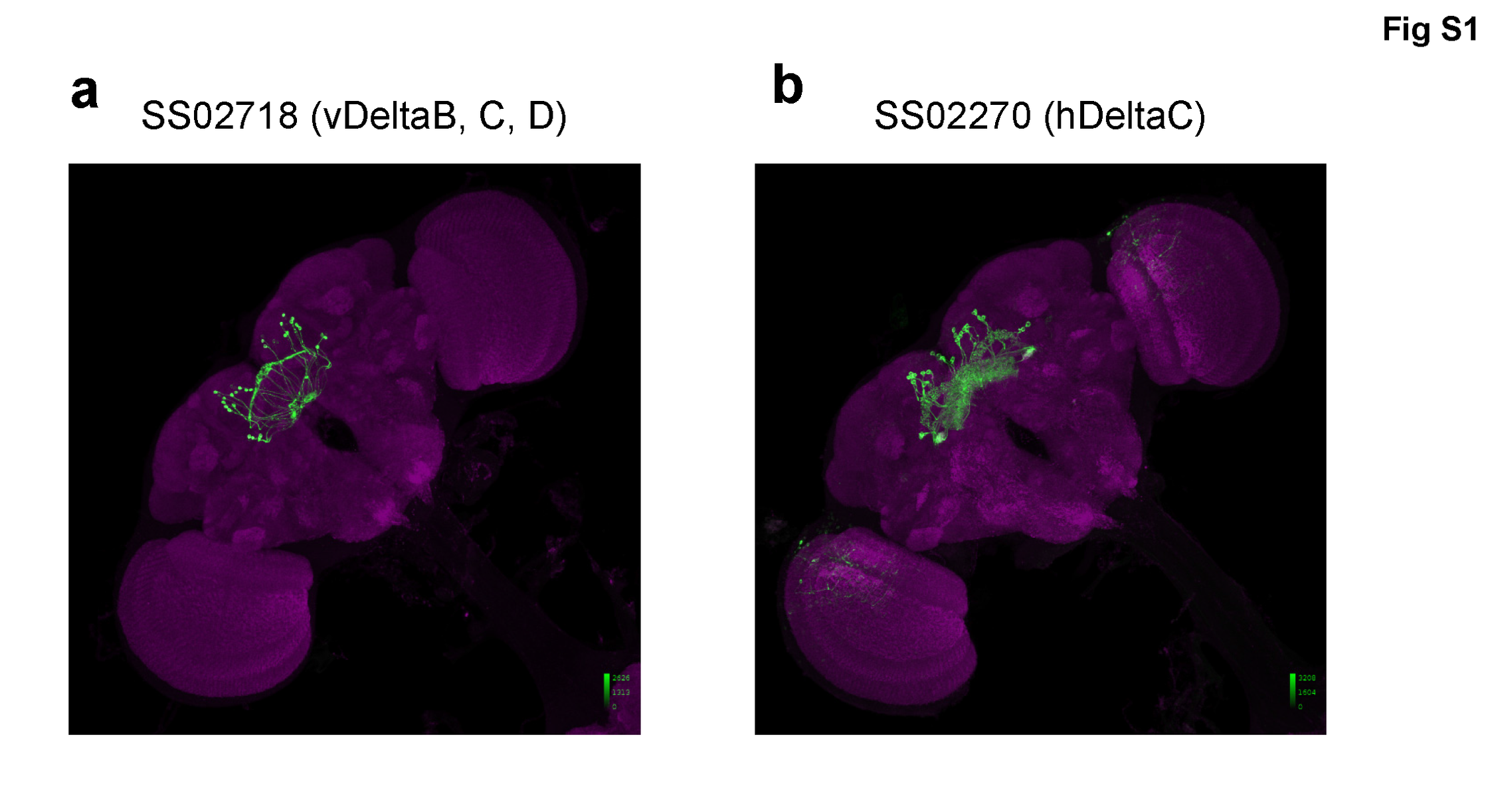

Supplement: S1 Fig — (a) Expression pattern of SS02718 and (b) SS02270 (from Janelia FlyLight Split-GAL4 Driver Collection). (TIF) [file pone.0277918.s001.tif]
